# Functioning among persons with lower limb amputation with or without prostheses in Rwanda

**DOI:** 10.4102/ajod.v12i0.1193

**Published:** 2023-10-17

**Authors:** Robert Ngarambe, Jean Baptiste Sagahutu, Assuman Nuhu, David K. Tumusiime

**Affiliations:** 1 Department of Physiotherapy, Faculty of Health Sciences, University of Rwanda, Kigali, Rwanda; 2 Department of Rehabilitation, Centre of Excellence in Biomedical Engineering and e-health, University of Rwanda, Kigali, Rwanda

**Keywords:** functioning, disability, mobility, participation, persons with lower limb amputation, prosthesis

## Abstract

**Background:**

Limb loss limits functioning and restricts participation in various environments. Persons with lower limb amputations (PLLA) experience challenges ranging from self-care and independence to psychological disorders that negatively impact their functioning.

**Objectives:**

To assess the functioning and the level of disability of PLLA with or without prostheses in Rwanda.

**Method:**

A descriptive, cross-sectional study was conducted among PLLAs aged 18 years and above in 10 districts of Rwanda. A total of 247 participants were purposively selected to fill the questionnaires. Descriptive and inferential statistics using *t*-test and binary logistic regression were performed to analyse data using Statistical Package for Social Sciences (SPSS) (version 21.0).

**Results:**

Out of 247 PLLA, 99 (40.1%) had prostheses and remaining 148 (59.9%) did not. Majority of PLLA without prostheses reported having more difficulties in mobility (s.d. 3.98), participation (s.d. 5.18) and life activities (s.d. 3.87). The majority of PLLA reported mild and moderate functioning in the domains of cognitive (odds ratio [OR] 8.842, 5.384 with 95% confidence interval [CI]) mobility (OR 16.154, 2.485 with 95% CI) and participation (OR 13.299, 15.282 with 95% CI).

**Conclusion:**

Persons without prostheses demonstrated reduced level of functioning and high levels of disability compared to those with prostheses in all domains. However, the mobility, self-activities and the participation domains were the mainly affected.

**Contribution:**

The study helps to understand the needs of the PLLA and emphasises that not only having prostheses can improve functioning but also emphasises the psychosocial aspects to reduce disability.

## Introduction

Persons with lower limb amputation (PLLA) experience reduced mobility, which leads to a number of difficulties ranging from self-care, independence and psychological well-being (Alessa et al. 2022). Lower limb amputation (LLA) highly leads to physical disability because of the loss of a body part (limb) that limits activity and restricts participation in various environments (Kostanjsek 2011). Decline in functioning leads to increase disability among persons with LLA and is further exacerbated by limited access to prostheses in low-middle income countries (Mattick et al. 2022). Although persons with LLA may use other assistive technologies (AT), prostheses are the most appropriate for mobility for an optimum functioning (Batten et al. 2019).

In low-income countries such as Rwanda, the availability, accessibility and affordability of prostheses are challenges. Therefore, without prostheses, the functioning and well-being of persons with LLA are highly affected (De Witte et al. 2018). With limited functioning, PLLA face challenges in education or finding employment, thus being among the most vulnerable in the society.

Without employment and a source of income, PLLA become a burden to the family and society in general (Van Twillert et al. 2014). Improved functioning of persons with LLA can be enhanced through the use of prostheses, in addition to other rehabilitation interventions such as gait training and improving mental health to reduce the level of disability (Batten et al. 2019).

The livelihood of persons with LLA may depend on having prostheses to enable them to contribute dynamically and effectively in their community, as well as to engage in income-generating activities (Zidarov, Swaine & Gauthier-Gagnon 2009). The disability of persons with LLA is not only caused by reduced functioning as a result of limb loss, but may rather be aggravated by both environmental and personal factors (Biggeri et al. 2014). Environmental factors such as a lack of basic infrastructure are the major hindrance to the use of prostheses in low- and middle-income countries; hence, reduced functioning. More so, individual factors such as psychological and emotional stress caused by the loss of limb are also key factors in functioning and participation (Von Kaeppler et al. 2021). The use of prostheses by persons with LLA is associated with a comprehensive rehabilitation programme which is aimed ultimately towards achieving functioning independence within the psychosocial, physical, as well as vocational aspects (Järnhammer et al. 2018; Layton & Steel 2015; Van Twillert et al. 2014).

In Rwanda, not much is known about the level of disability and functioning of person with LLA The aim of the study therefore was to compare the levels of functioning and disability in people with and without prostheses in Rwanda.

## Research methods and design

The study was conducted in 10 out of 30 districts in the 4 provinces and the city of Kigali in Rwanda. A random sampling of two districts were selected from the four provinces and the city of Kigali in Rwanda. These were Rusizi and Nyamashake in the western province, Huye and Nyanza in the southern province, Musanze and Rulindo in the northern province, Kayonza and Kirehe in the eastern province, and Kicukiro and Nyarugenge in the city of Kigali. A cross-sectional, descriptive study design was used to assess the functioning and disability levels of persons with LLA. The population sample was retrieved from the database of the National Council of People with Disabilities (NCPD) in the 10 districts which was 362 persons with LLA. The NCPD is a government agency gazetted by law to oversee all activities of persons with disability in Rwanda. A total of 247 persons with LLA participated in the study from the 362 persons with LLA in the 10 districts. The study included participants with LLA who were 18 years and above, and excluded PLLA with spinal cord injuries or any other lower limb impairments that might impact on the use of prostheses. The participants in the study were recruited through the NCPD at the district; thereafter, the research assistant contacted the participants from their villages and met them at the sector administration office for data collection. The researcher explained in detail the purpose of the study to the participants, requested their voluntary participation, and then collected information from them by filling the questionnaires, while addressing and clarifying any concerns the participants had about the questionnaire. The research assistant also administered the questionnaires to participants who did not know how to read and write.

The 36-item World Health Organization Disability Assessment Schedule 2.0 (WHODAS 2.0) has been endorsed by the World Health Organization (WHO) to measure physical, mental, social and functioning disability (Üstün et al. 2010). The WHODAS 2.0 assesses the functioning and disability in six domains – cognitive, mobility, self-care, getting along, daily life activities and participation in society (Garin et al. 2010). The domains assess different dimensions of activities in communication and thinking activities, movement challenges, taking care of oneself, socialising with others, difficulties in the everyday activities and difficulties in participation in society. The WHODAS 2.0 version follows a five-point Likert scale (0 = none, 1 = mild, 2 = moderate, 3 = severe, 4 = extreme/cannot do anything) regarding difficulties for each item faced in the past 30 days (available from the WHO website: https://www.who.int/standards/classifications/international-classification-of-functioning-disability-and-health/who-disability-assessment-schedule). The WHODAS 2.0 has proved to be reliable since it has been used and compared in different context including Sub-Saharan African countries like Rwanda among children, Ethiopia and Tanzania (Cronbach alpha = 0.82) (Habtamu et al. 2017; Mwanyangala et al. 2010; Scorza et al. 2013; Silveira et al. 2013). The validity is also high since it has been validated in both low- and high-income countries, and has shown a high concurrent validity among specific domain correlations after concurrent administration in comparison to the International Classification of Functioning (ICF) domain (Üstün et al. 2010).

The questionnaire was translated from English language to Kinyarwanda language. Both forward and backward translations were performed by two professional translators to address the cultural and linguistic equivalence. Regarding the opinion on the clarity, quality of translation and suitability of the study, the questionnaire was sent to two specialists in the field of rehabilitation.

The study was approved for the ethical clearance by the Institution Review Board (IRB) of the University of Rwanda, College of Medicine and Health Sciences; N°369/CMHSIRB/2020. Permission was obtained from the NCPD N°485/NCPD/2021. Permission was granted by the district authorities to meet persons with LLAs from their communities.

Descriptive statistics were performed to summarise the demographic data using Statistical Package for Social Sciences (SPSS) (version 21.0). The WHODAS domain scores were summed to overall WHODAS score, then transformed into a 0–100 scale, with 0 representing no disability and 100 representing the highest disability. The chi-square was performed to determine the association between persons with LLA with or without prostheses and demographic data. The *t*-test was performed to compare means of functioning between persons with and without prostheses.

Binary logistic regression analysis was carried out to determine the association between persons with or without prostheses and functioning domains among participants. The level of significance was set at (*p* < 0.05).

### Ethical considerations

The study was approved for the ethical clearance by the Institution Review Board (IRB) of the University of Rwanda, College of Medicine and Health Sciences with (ethical clearance number N°369/CMHSIRB/2020). Permission was obtained from the NCPD; N°485/NCPD/2021. Permission was granted by the district authorities to meet persons with LLAs from their communities. Signed informed consent was obtained from all individual participants.

## Results

### Sociodemographic characteristics of persons with lower limb amputations

A total number of 247/362 (68.2%) persons with LLA participated in this study from the 10 selected districts of Rwanda. The low turnout may have been as a result of the inclusion and exclusion criteria or participants felt not comfortable to participate in the study. The age of the participants ranged from 18 to 79 years, with a mean age of 43.4 years (standard deviation [s.d.] = 14.1). Among the participants, males accounted for 171/247 (69.2%) and females 76/247 (30.8%). The characteristics of the participants are highlighted in Table 1. Among the participants, 109/247 (44.1%) males and 39/247(15.8%) female did not have prostheses. There was no statistically significant association between gender and possession of prostheses among persons with LLA (*p* = 0.066). Among the participants, the 38–47 year age group had the most prostheses at 25/247 (10.1%), followed by the 28–37 age group at 24/247 (9.7%). Majority 166/247 (67.2%) of persons with LLA lived in the rural area, of which the 95/247 (38.5%) did not have any prostheses, while among the participants in urban areas 28/247(11.3%) had prostheses.

**TABLE 1:** Sociodemographic characteristics of persons with lower limb amputations.

Variables	Characteristics	Having prosthesis						
		No		Yes		Total		Statistics
		Number	%	Number	%	Number	%	
Sex	Male	109	44	62	25	171	69.2	0.066
	Female	39	16	37	15	76	30.8	-
Age	18–27	22	8.9	11	4.5	33	13.4	0.124
	28–37	40	16	24	9.7	64	25.9	-
	38–47	31	13	25	10	56	22.7	-
	48–57	31	13	22	8.9	53	21.5	-
	58–67	9	3.6	13	5.3	22	8.9	-
	68 and above	15	6.1	4	1.6	19	7.7	-
Level of amputation	Transtibial	76	31	50	20	126	51	0.627
	Transfemoral	68	28	44	18	112	45.3	-
	Bilateral amputation	4	1.6	5	2	9	3.6	-
Education	Never went to school	31	13	19	7.7	50	20.2	0.086
	Primary	80	32	44	18	124	50.2	-
	High school	31	13	25	10	56	22.7	-
	Technical/Vocational	5	2	5	2	10	4	-
	University	1	0.4	6	2.4	7	2.8	-
Employment	Employed	3	1.2	10	4	13	5.3	0.062
	Self-employed	58	24	39	16	97	39.3	-
	Volunteer	3	1.2	2	0.8	5	2	-
	Student	5	2	2	0.8	7	2.8	-
	Housekeeping	10	4	9	3.6	19	7.7	-
	Retired	5	0	0	0	5	2	-
	Unemployed	64	26	37	15	101	40.9	-
Marital status	Single	30	12	26	11	56	22.7	0.576
	Married	74	30	53	22	127	51.4	-
	Separated	15	6.1	6	2.4	21	8.5	-
	Divorced	4	1.6	1	0.4	5	2	-
	Widowed	6	2.4	4	1.6	10	4	-
	Cohabiting	19	7.7	9	3.6	28	11.3	-
Residence	Urban	53	22	28	11	81	32.8	0.217
	Rural	95	39	71	29	166	67.2	-

Chi-square, (*p* < 0.05).

### Function and disability scores among persons with lower limb amputations with or without prostheses

The results indicated that persons with LLA without prosthesis significantly scored generally higher disability levels (mean 84.32, s.d. 15.67) compared to persons with prosthesis (mean 62.03, s.d. 12.34) (Table 2). Participants without prostheses were more affected in the physical domains such as participation in society domain (mean 21.14, s.d. 5.18), mobility domain (mean 19.76, s.d. 3.98) and life activities domain (mean 19.50, s.d. 3.22) as compared to persons with prostheses in the same domains of society (mean 18.12, s.d. 3.74), mobility (mean 16.00, s.d. 3.21) and life activities (mean 15.97, s.d. 3.22). Although persons with LLA reported more difficulties in mobility, life activities and participation, the psychosocial domains such as the understanding and communication and getting along with people domains were equally affected. However, the results further portrayed that there was significant change between participants with prostheses in the domains of understanding and communication (mean 2.04, s.d. 1.62), getting along with people (mean 5.34, s.d. 2.55) and participants without prostheses in the same domains of understanding and communication (mean 6.35, s.d. 3.97) and getting along with people domains (mean 8.36, s.d. 3.11). There was a statistically significant difference in all domains between participants having or not having prostheses (*p* < 0.001).

**TABLE 2:** Functioning domain mean scores of persons with lower limb amputations with or without prostheses.

Domain	Having a prosthesis				
	No (*n* = 148)		Yes (*n* = 99)		Statistics
	Mean	s.d.	Mean	s.d.	*df* = 245
Understanding and communicating	6.35	3.97	2.04	1.62	*t* = 10.24*
Mobility	19.76	3.98	16.00	3.21	*t* = 7.40*
Self-care	9.21	3.13	4.35	2.63	*t* = 13.11*
Getting along with people	8.36	3.11	5.34	2.55	*t* = 7.35*
Life activities	19.50	3.87	15.97	3.22	*t* = 8.80*
Participation in society	21.14	5.18	18.12	3.74	*t* = 4.98*
General disability score	84.32	15.67	62.03	12.34	*t* = 11.89*

* , *p* < 0.001.

s.d., standard deviation; *t*, T. test; *df,* degree of freedom.

### Functioning and disability among persons with lower limb amputations

The study findings indicated that persons with prostheses in employment had 15 times more chances of better functioning compared to the persons without prostheses (odds ratio [OR] 15.477 with 95% confidence interval [CI] of 1.265 to189.83). The difference in functioning within employment was seen in other categories also such as self-employed, volunteers, students and house-keeping among PLLA with or without prostheses.

The study findings show that having a prosthesis among participants had more chances of better functioning among the understanding and communication domain (OR 8.842 with a 95% CI of 1.041 to 75.140, 5.384 with a 95% C.I of 0.461 to 62.840). The results from the study revealed that functioning increased in the mobility domain among participants with prostheses (OR 16.154 with a 95% CI of 5.595 to 46.637, 2.485 with a 95 %CI of 1.009 to 6.118) (see Table 3). There was statistically significant association between having prostheses and level of functioning in mobility domain (*p* < 0.001).

**TABLE 3:** Logistic regression of functioning in domains of persons with lower limb amputations.

Variables	Characteristics	OR	95% CI		*p*
			Lower	Upper	
Sex	Male	0.595	0.242	1.463	0.258
	Female	1	-	-	-
Age	18–27	0.082	0.005	1.343	0.08*
	28–37	0.169	0.014	2.099	0.166
	38–47	0.029	0.023	3.68	0.34*
	48–57	0.32	0.03	3.422	0.346
	58–67	0.339	0.302	32.554	0.339
	68 + (reference)	1	-	-	-
Employment	Employed	15.48	1.265	189.83	0.032*
	Self-employed	2.091	0.856	5.108	0.106
	Volunteer	3.564	0.255	49.778	0.345
	Student	3.384	0.398	28.736	0.264
	Housekeeping	1.457	0.289	7.331	0.648
	Retired	0	0	-	0.999
	Unemployment (reference)	1	-	-	-
Level of amputation	Transtibial amputation	0.033	0.002	0.615	0.022*
	Transfemoral amputation	0.042	0.003	0.601	0.020*
	Bilateral amputation (reference)	1	-	-	-
Residence	Urban	0.283	0.119	0.671	0.004*
	Rural (reference)	1	-	-	-
Domains	**Cognitive**	-	-	-	0.112
	Mild	8.842	1.041	75.14	0.046*
	Moderate	5.384	0.461	62.84	0.179
	Severe (reference)	-	-	-	-
	Mobility	-	-	-	<0.001*
	Mild	16.154	5.595	46.637	<0.001*
	Moderate	2.485	1.009	6.118	0.048*
	Severe (reference)	-	-	-	-
	Self-care	-	-	-	0.17
	Mild	0.298	0.085	1.051	0.06
	Moderate	0.343	0.071	1.665	0.184
	Severe (reference)	-	-	-	-
	Getting along with people	-	-	-	0.369
	Mild	0.684	0.238	1.965	0.481
	Moderate	0.375	0.092	1.526	0.171
	Severe (reference)	-	-	-	-
	Life activities	-	-	-	0.689
	Mild	0.432	0.023	8.105	0.574
	Moderate	0.256	0.009	7.055	0.421
	Severe (reference)	-	-	-	-
	Participation in society	-	-	-	0.031*
	Mild	13.299	1.889	93.648	0.009*
	Moderate	15.282	1.841	126.879	0.012*
	Severe (reference)	-	-	-	-
	Overall score	-	-	-	0.37
	Mild	3.538	0.535	23.395	0.19
	Moderate	2.412	0.262	22.249	0.437
	Severe (reference)	-	-	-	-
	Constant	0.007	-	-	0.017

Note: Dependent Variable: Prosthesis: 1 Having prosthesis, 0 not having prosthesis.

CI = confidence interval, OR = odds ratio, *p* < 0.05.

* , statistically significant.

The study findings indicated that persons with prostheses had increased functioning in the participation domain compared to the persons without prostheses (OR 13.299 with a 95% CI of 1.889 to 93.648, 15.282 with a 95% CI of 1.841 to 126.879. There was a statistically significant association between having prostheses and the level of functioning in participation in society domain (*p* = 0.031).

The results further underscored that the level of functioning among participants with prostheses was more likely not to change in the self-care domains.

The findings from the study indicated that the level of disability from the overall score showed that participants with prostheses had mild disability (OR 3.538 with a 95% CI of 0.535 to 23.395 and moderate disability at OR 2.412 with a 95% CI of 0.262 to 22.249).

## Discussion

The study assessed the functioning and disability levels of persons with LLA with or without prostheses in Rwanda. Persons with LLA are considered as having limited functioning because of the loss of body structure; hence, increased levels of disability (Ng et al. 2020). The study findings highlighted that 148/247 (59.9%) of the participants did not have prostheses. The findings are similar to the studies in low- and middle-income countries (LMICs) that hinted at a gap in the provision of assistive technologies (Visagie et al. 2017). This evidently means with the low provision of prostheses to PLLA, persons without prostheses will have minimum functioning henceforth causing disability (Von Kaeppler et al. 2021). Furthermore, Wyss et al. (2015) argued that limited access to prostheses in LMICs is largely because of the lack of components such as knees and feet for fabricating the prosthesis. In addition, limited training of professionals in prosthetic fabrication and limited resources may also contribute to the inaccessibility (Järnhammer et al. 2018).

Among the participants of this study, the age groups 28 years–37 years and 38 years–47 years had the most prostheses than the rest of other age groups and was followed by the 48 years–57 years age group. Persons with LLA in these age groups may have more access to prostheses because of the fact that they have families to take care of, and therefore have to find a way of improving their mobility to take care of their families through finding employment or other source of income to improve their well-being. As reported in a study conducted in India on the quality of life among persons with LLA, prosthetic use increases the chances of employment hence improving livelihood (Sinha, Van Den Heuvel & Arokiasamy 2011). More so, the same observation has been underscored in a systemic review by Hunt et al. (2022) on the effectiveness of interventions to improve the livelihood of persons with disabilities. Therefore, more efforts are needed to improve access to prostheses in other age groups in LMICs which will lead to increasing level of functioning and resultantly reducing the level of disability.

The results of this study indicated that the majority of participants without prostheses were unemployed against the majority of participants with prostheses. However, this also means that without a prosthesis it is hard to engage in income-generating activities, can thus it is hard to afford to prostheses. The findings of this study are similar to a study done in Nepal, which indicated that though persons with LLA had prostheses, they still had difficulties using the devices because of poor prosthetic technology that could enable them to use prostheses to walk and work in the challenging landscape of the mountainous regions of western Nepal (Järnhammer et al. 2018) which is similar to that in Rwanda.

The findings from this study further accentuated that participants with prostheses were more likely to be employed than those without prostheses, which is likely the result of increased functioning afforded by having a prosthesis. The results concur with the findings from the study conducted among patients with schizophrenia, and this severe disability was reported to contribute to their unemployment because of limited functioning (Lu et al. 2018). Furthermore, the results from this study revealed that participants with prostheses had improved functioning and low levels of disability. Von Kaeppler et al. (2021) underscored that having prostheses tremendously improves functioning and reduces the severity of disability through enhanced mobility.

The findings from this study indicated severity of disability did not depend on either gender or place of residence when using prostheses. However, mobility, life activities and participation in society were dependent on having or not having prostheses among persons with LLA in this study. These findings concur with the study done in Bangladesh among people with spinal cord injuries that reported environmental and physical barriers that often contributed to activity limitations and participation restrictions, more especially in rural areas (Kader 2018).

Findings from this study also revealed that the female participants were more likely to have high level of difficulties in functioning than the male participants. The results from this study agree with a study done in Tanzania that found that men had less physical disability compared to women (Mwanyangala et al. 2010). Moreover, the findings from this study have revealed that older persons with LLA hardly had prostheses compared to the younger ones, and they are therefore likely to have reduced functioning and increased levels of disabilities. The results are in agreement with a study done on persons with LLA in India by Sihna et al. (2011), who reported that older age and comorbidity were a greater hindrance to functioning and therefore increased levels of disability. Although the findings in this study show that severity of disability and reduced functioning among persons with LLA may be attributed to age and gender since majority of older and female participants had more disability, this may not be true because these findings differ from a study conducted in LMICs – South Africa, Ghana Indonesia, Tanzania, Kenya, Bangladesh, India and Vietnam – that did not show any relationship between level of disability, age and gender. Though the authors concluded that such a relationship may depend on individual countries, the level of disability is the same across all genders and sociocultural context (Gomez-Olive et al. 2017).

The study findings uncovered that participants had less difficulties in the three domains (cognitive, self-care and getting along with people) than the domains of mobility, life activities and the participation in society. Furthermore, the cognitive domain was reported to have the least participants with impairments. This signifies that amputation, being a physical impairment, may not severely affect the mental aspect. However, some may be affected at a certain degree as revealed in this study.

The current results differ from the study conducted by Amosun, Mutimura and Frantz (2005) that disclosed that the persons with LLA had emotional effects that resulted into secondary level of disabilities, and that further led them into more dependence. The results in study further emphasised that participants in the domains of mobility, life activity and participation in society have more reduced functioning and higher disability levels.

Literature underscores that limitation to functioning is a result of both intrinsic factors which are directly from the individual and extrinsic factors which are from the environment (Reitzel et al. 2021). Although this study was conducted in a different setting with different characteristics, the findings were similar to the study conducted by Gallagher et al. (2011) which reported that environmental barriers are among the restrictions to participation and hence affecting these domains.

Furthermore, the study results pointed out that persons with prostheses had less difficulties in the mobility and participation in society domains than their counterparts without prostheses. The results are in agreement with the study done in Tanzania that stressed that provision of prostheses improves the functioning as well as the quality of life (Von Kaeppler et al. 2021) More so, a systematic review done by Davie-smith et al. (2017) agrees with the findings in this study that provision of assistive technology such as prostheses may improve activity limitation and participation restriction of persons with LLA.

Prostheses alone cannot improve functioning and reduce the level of disabilities unless personal, environmental and infrastructural challenges are considered to facilitate the mobility of persons with LLA in both private and public areas. It is in this regard that the Rwandan government enacted policies to improve the accessibility of prostheses such as law on subsidising the cost of healthcare through community health insurance where persons with LLA can get prostheses at subsidised cost (Kidd & Kabare 2019).

### Implications

The study findings help to understand the needs of the persons with LLA with or without prostheses. The findings emphasise not only the importance of having prostheses but also that environmental and infrastructural barriers should be well-thought-of during the rehabilitation process in order to improve the functioning. The inclusion of psychosocial rehabilitation of persons with LLA is considered to improve functioning and reduce disability. Thus, initiatives that focus on improving functioning and general welfare of persons with LLA are recommended.

### Limitations

This study may be the first to assess the functioning of persons with LLA in Rwanda. The WHODAS 2.0 questionnaire primarily measures functioning and disability, yet persons with LLA may have diverse disabilities that may not all be exhausted with WHODAS 2.0. However, it was more suitable since the study was mostly looking at functioning of persons with LLA. Another limitation was that the study being a cross-sectional design may not have exhausted all causes that limits the functioning of persons with LLA. A longitudinal design that would follow up with the participants for some time to exhaust the challenges to functioning, was likely the best approach. It is also possible that prostheses may have increased functioning and reduced the levels of disability of the participants. Lastly, the study did not find out from the participants if there were other comorbidity factors that could influence functioning of persons with LLA. Comorbidity factors such as illnesses like diabetes may weaken the body and hence limit the functioning. Further research is therefore needed to examine the influence of comorbidities on persons with or without prostheses.

## Conclusion

Persons without prostheses demonstrated reduced level of functioning and high levels of disability compared to those with prostheses in all domains. The mobility, self-activities and the participation domains were mainly affected. However, the psychosocial domains were equally affected and PLLA with prostheses were less affected more so, gender and advancing age were highlighted to increase difficulties in functioning and disability among persons with LLA.

## References

[CIT0001] Alessa, M., Alkhalaf, H.A., Alwabari, S.S., Alwabari, N.J., Alkhalaf, H., Alwayel, Z. et al., 2022, ‘The psychosocial impact of lower limb amputation on patients and caregivers’, *Cureus* 14(11), e31248. 10.7759/cureus.31248 36505108 PMC9731396

[CIT0002] Amosun, S.L., Mutimura, E. & Frantz, J.M., 2005, ‘Health promotion needs of physically disabled individuals with lower limb amputation in Rwanda’, *Disability and Rehabilitation* 27(14), 837–847. 10.1080/09638280400018676 16096236

[CIT0003] Batten, H.R., McPhail, S.M., Mandrusiak, A.M., Varghese, P.N. & Kuys, S.S., 2019, ‘Gait speed as an indicator of prosthetic walking potential following lower limb amputation’, *Prosthetics and Orthotics International* 43(2), 196–203. 10.1177/0309364618792723 30112982

[CIT0004] Biggeri, M., Deepak, S., Mauro, V., Trani, J.-F., Kumar, J. & Ramasamy, P., 2014, ‘Do community-based rehabilitation programmes promote the participation of persons with disabilities? A case control study from Mandya District, in India’, *Disability and Rehabilitation* 36(18), 1508–1517. 10.3109/09638288.2013.823244 23944177

[CIT0005] Davie-Smith, F., Coulter, E., Kennon, B., Wyke, S. & Paul, L., 2017, ‘Factors influencing quality of life following lower limb amputation for peripheral arterial occlusive disease: A systematic review of the literature’, *Prosthetics and Orthotics International* 41(6), 537–547. 10.1177/0309364617690394 28147898

[CIT0006] De Witte, L., Steel, E., Gupta, S., Ramos, V.D. & Roentgen, U., 2018, ‘Assistive technology provision: Towards an international framework for assuring availability and accessibility of affordable high-quality assistive technology’, *Disability and Rehabilitation: Assistive Technology* 13(5), 467–472. 10.1080/17483107.2018.1470264 29741965

[CIT0007] Gallagher, P., O’Donovan, M., Doyle, A. & Desmond, D., 2011, ‘Environmental barriers, activity limitations and participation restrictions experienced by people with major limb amputation’, *Prosthetics and Orthotics International* 35(3), 278–284. 10.1177/0309364611407108 21937573

[CIT0008] Garin, O., Ayuso-Mateos, J.L., Almansa, J., Nieto, M., Chatterji, S., Vilagut, G. et al., 2010, ‘Validation of the “World Health Organization Disability Assessment Schedule, WHODAS-2” in patients with chronic diseases’, *Health and Quality of Life Outcomes* 8, 51. 10.1186/1477-7525-8-51 20482853 PMC2893517

[CIT0009] Gomez-Olive, F.X., Schröders, J., Aboderin, I., Byass, P., Chatterji, S., Davies, J.I. et al., 2017, ‘Variations in disability and quality of life with age and sex between eight lower income and middle-income countries: Data from the INDEPTH WHO-SAGE collaboration’, *BMJ Global Health* 2(4), 1–11. 10.1136/bmjgh-2017-000508 PMC5759706 29333288

[CIT0010] Habtamu, K., Alem, A., Medhin, G., Fekadu, A., Dewey, M., Prince, M. et al., 2017, ‘Validation of the World Health Organization Disability Assessment Schedule in people with severe mental disorders in rural Ethiopia’, *Health and Quality of Life Outcomes* 15(1), 1–11. 10.1186/s12955-017-0647-3 28381230 PMC5382515

[CIT0011] Hunt, X., Saran, A., Banks, L.M., White, H. & Kuper, H., 2022, ‘Effectiveness of interventions for improving livelihood outcomes for people with disabilities in low- and middle-income countries: A systematic review’, *Campbell Systematic Reviews* 18(3), e1257. 10.1002/cl2.1257 36913195 PMC9246293

[CIT0012] Järnhammer, A., Andersson, B., Raj Wagle, P. & Magnusson, L., 2018, ‘Living as a person using a lower-limb prosthesis in Nepal’, *Disability and Rehabilitation* 40(12), 1426–1433. 10.1080/09638288.2017.1300331 28320228

[CIT0013] Kader, M., 2018, ‘Socio-demographic and injury-related factors contributing to activity limitations and participation restrictions in people with spinal cord injury in Bangladesh’, *Spinal Cord* 56, 239–246. 10.1038/s41393-017-0001-y 29093546

[CIT0014] Kidd, S. & Kabare, K., 2019, *Social protection and disability in Rwanda*, pp. 1–56, viewed 23 April 2020, from www.developmentpathways.co.uk.

[CIT0015] Kostanjsek, N., 2011, ‘Use of the International Classification of Functioning, Disability and Health (ICF) as a conceptual framework and common language for disability statistics and health information systems’, *BMC Public Health* 11(suppl 4), 2–7. 10.1186/1471-2458-11-S4-S3 21624189 PMC3104216

[CIT0016] Layton, N.A. & Steel, E.J., 2015, ‘“An environment built to include rather than exclude me”: Creating inclusive environments for human well-being’, *International Journal of Environmental Research and Public Health* 12(9), 11146–11162. 10.3390/ijerph120911146 26371024 PMC4586666

[CIT0017] Lu, S.J., Liou, T.H., Yen, C.F., Chang, F.-H., Chen, Y.-L., Escorpizo, R. et al., 2018, ‘Determinants of employment outcome for the people with Schizophrenia using the WHODAS 2.0’, *Journal of Occupational Rehabilitation* 29, 375–383. 10.1007/s10926-018-9794-6 29951935

[CIT0018] Mattick, K., Oldfrey, B., Donovan-Hall, M., Magomere, G., Gakunga, J. & Holloway, C., 2022, ‘Experiences of lower limb prosthesis users in Kenya : A qualitative study to understand motivation to use and satisfaction with prosthetic outcomes ABSTRACT’, *Disability and Rehabilitation* 0(0), 1–11. 10.1080/09638288.2022.2152875 36495104

[CIT0019] Mwanyangala, M., Mayombana, C., Urassa, H., Charles, J., Mahutanga, C., Abdullah, S. et al., 2010, ‘Health status and quality of life among older adults in rural Tanzania’, *Global Health Action* 3(1), 2142. 10.3402/gha.v3i0.2142 PMC2958089 20975983

[CIT0020] Ng, S.S., Naing, L., Idris, F.I. & Pande, K., 2020, ‘What is the quality of life of transtibial amputees in Brunei Darussalam?’, *Malaysian Orthopaedic Journal* 14(2), 39–46. 10.5704/MOJ.2007.009 32983376 PMC7513655

[CIT0021] Reitzel, M., Letts, L., Di Rezze, B. & Phoenix, M., 2021, ‘Critically examining the person – Environment relationship and implications of intersectionality for participation in children’s rehabilitation services’, *Frontiers in Rehabilitation Sciences* 2, 10–12. 10.3389/fresc.2021.709977 PMC9397911 36188778

[CIT0022] Scorza, P., Stevenson, A., Canino, G., Mushashi, C., Kanyanganzi, F., Munyanah, M. et al., 2013, ‘Validation of the “World Health Organization Disability Assessment Schedule for Children, WHODAS-Child” in Rwanda’, *PLoS One* 8(3), e57725. 10.1371/journal.pone.0057725 23505437 PMC3591425

[CIT0023] Silveira, C., Parpinelli, M.A., Pacagnella, R.C., De Camargo, R.S., Costa, M.L., Zanardi, D.M. et al., 2013, ‘Cross – Cultural adaptation of the World Health Organization’, *Revista da Associação Médica Brasileira* 58(3), 234–240. 10.1016/S2255-4823(13)70462-4 23684209

[CIT0024] Sinha, R., Van Den Heuvel, W.J.A. & Arokiasamy, P., 2011, ‘Factors affecting quality of life in lower limb amputees’, *Prosthetics and Orthotics International* 35(1), 90–96. 10.1177/0309364610397087 21515894

[CIT0025] Üstün, T.B., Chatterji, S., Kostanjsek, N., Rehm, J., Kennedy, C., Epping-Jordan, J. et al., 2010, ‘Developing the World Health Organization disability assessment schedule 2.0’, *Bulletin of the World Health Organization* 88(11), 815–823. 10.2471/BLT.09.067231 21076562 PMC2971503

[CIT0026] Van Twillert, S., Stuive, I., Geertzen, J.H.B., Postema, K. & Lettinga, A.T., 2014, ‘Functional performance, participation and autonomy after discharge from prosthetic rehabilitation: Barriers, facilitators and outcomes’, *Journal of Rehabilitation Medicine* 46(9), 915–923. 10.2340/16501977-1846 25074343

[CIT0027] Visagie, S., Eide, A.H., Mannan, H., Schneider, M., Swartz, L., Mji, G. et al., 2017, ‘A description of assistive technology sources, services and outcomes of use in a number of African settings’, *Disability and Rehabilitation: Assistive Technology* 12(7), 705–712. 10.1080/17483107.2016.1244293 27882821

[CIT0028] Von Kaeppler, E.P., Hetherington, A., Donnelley, C.A., Ali, S.H., Shirley, C., Challa, S.T. et al., 2021, ‘Impact of prostheses on quality of life and functional status of transfemoral amputees in Tanzania’, *African Journal of Disability* 10, 1–10. 10.4102/ajod.v10i0.839 PMC8517763 34692432

[CIT0029] Wyss, D., Lindsay, S., Cleghorn, W. & Andrysek, J., 2015, ‘Priorities in lower limb prosthetic service delivery based on an international survey of prosthetists in low- and high-income countries’, *Prosthetics and Orthotics International* 39(2), 102–111. 10.1177/0309364613513824 24335154

[CIT0030] Zidarov, D., Swaine, B. & Gauthier-Gagnon, C., 2009, ‘Life habits and prosthetic profile of persons with lower-limb amputation during rehabilitation and at 3-month follow-up’, *Archives of Physical Medicine and Rehabilitation* 90(11), 1953–1959. 10.1016/j.apmr.2009.06.011 19887223

